# Supplementary material for: NeuroMotion: Open-source platform with neuromechanical and deep network modules to generate surface EMG signals during voluntary movement
Source: PLoS Comput Biol. 2024 Jul 3;20(7):e1012257. doi: 10.1371/journal.pcbi.1012257 (PMC11251629; doi:10.1371/journal.pcbi.1012257)
Supplement: Attachment — Submitted filename: response_letter.pdf [file pcbi.1012257.s001.pdf]

Response to Reviewers' Comments

Dear Editors of PLOS Computational Biology,

We are very grateful for the suggestions and comments from the editors and reviewers concerning our manuscript entitled "NeuroMotion: Open-Source Simulator to Generate High-Density EMG Signals during Voluntary Movement" (PCOMPBIOL-D-23-01615). We thank you most gratefully for the option to revise and resubmit this paper, an opportunity that we have taken advantage of with a thorough revision. We also thank the reviewers for the time they have taken to produce such an extensive and in-depth critique, which has greatly strengthened the final work. We have considered all comments very carefully and made corrections, substantial additional analyses, and explanations, which we hope will address the concerns and questions raised by the reviewers.

In the following, the comments from the reviewers are itemised for clarity and are addressed in order. The replies include references to the exact lines where the revisions to the text can be found. In the attached revised manuscript, all additions have been highlighted in yellow.

Reviewer 1

The authors proposed an innovative electromyographic generative model aimed at reproducing electrophysiological signals (BIOMIME) during dynamic contractions. The new model includes a previously developed model for the generation of motor unit action potentials during dynamic contractions, an OPENSIM muscular skeletal model for the simulation of muscle morphological parameters of the upper limb during dynamic movements, and a motor unit pool model to generate the spike trains. In general, the manuscript is well written and extremely important for the field, but the authors should improve the justification for their work. In particular, the innovation of the present work is not clear in comparison to the state-of-the-art. Additionally, some of the conclusions require further validation. Specifically:

Reviewer Point 1.1 *Validation results are reported for three subjects only, and no statistical analysis has been performed. The synthetic EMG signals have been tested to predict the joint angles of three subjects. The number of subjects is very limited, and the authors reported only*

the averaged correlation coefficients across different movements, making it difficult to evaluate the performance of the model. Specifically, it is complex to evaluate how good a PCC of 0.54 or 0.72 is without a comparison to anything else. These statistics also refer only to one subject, while for the other two, only the best regressors are mentioned, not the average among all DoF. A larger pool of subjects should be used, and a full statistical analysis of the results (average, std, distribution of the results, etc.) across all subjects should be presented. For example, in the manuscript, no information is presented about the results on the third subject, and in this specific case, it is rather important since it constitutes 1/3 of the validation dataset.

REPLY:

Thank you for noting this important omission. We have now added a detailed analysis of the experimental results for validating the potential applications of the proposed simulation platform. We have increased the number of subjects to six and provided a full analysis of the regression results, including the mean and standard deviation of the regression accuracy computed over the six subjects. We have also added one baseline as an estimation of the lower bound to account for the low-frequency property of the joint angles. Results showed that the Pearson correlation coefficient (PCC) between the predictions and the ground truth labels is significantly higher than this lower bound. Details of the implementation and the results can be found in the reply to point 1.8. We have now added these analyses and justifications to the Results at lines 393-465. We feel the resultant investigations have greatly strengthened the arguments in the revised manuscript.

We want to emphasise that this preliminary case study is a proof of concept that the synthetic data produced by NeuroMotion could be used for data augmentation, but not to demonstrate that the augmentation works for all subjects, given the difference between each subject's anatomy and the anatomy used in NeuroMotion. We have now added the limitations of NeuroMotion and their potential solutions to the Discussion in the revised manuscript. It has also to be noted that data augmentation, here presented as an example application, is not the only potential use of NeuroMotion. NeuroMotion can be used to provide datasets for testing adaptive EMG decomposition algorithms and studying the impact of physiological parameters on the output signals. We have added more details on these aspects to the Results in the revised manuscript so that the impact is more evident.

Reviewer Point 1.2 *Since the generation of the synthetic dataset is non-deterministic, multiple*

datasets could also be generated and tested, and the authors should check the consistency of different simulations.

REPLY:

This is an excellent point. We agree with the reviewer that the synthetic dataset is not deterministic under a specific movement. A primary reason is that motor unit spike trains are generated with natural variability in the interspike intervals [1]. To test the consistency of multiple simulation runs, we have now simulated the spike trains for one subject five times, i.e., five repeats for each of the five trials in each of the two degrees of freedom (DoFs). The spike trains were convolved with the motor unit action potentials (MUAPs), which were generated by BioMime by taking in the same latent representation and the physiological parameters that changed during the movement.

When we trained the regressor on the synthetic dataset generated during each run and tested the regressors to predict the joint angles of the experimental dataset, we found consistent regression results across the five simulation runs. The PCC values are normally distributed (Shapiro-Wilk test with $p > 0.05$) for both wrist (0.73 ± 0.01, mean $\pm$ std) and MCP (0.45 ± 0.07). We added a more detailed description of the simulation process to the Results to clarify this point.

Reviewer Point 1.3 *The results of the proposed model have not been compared to other state-of-the-art methods or models.*

REPLY:

Currently, there are no such forward models that simulate surface EMG signals during voluntary movement while providing all intermediate variables, including the spike trains, MUAPs, and physiological parameters. The proposed simulation platform is indeed the first that could be used to simulate surface EMG signals in high temporal resolution while explicitly providing all input, intermediate, and output variables. There have been methods that simulate muscle activations given kinematics, which we have discussed in the Introduction, but these are not structural models and can only simulate the envelopes of sEMG signals. We have now improved our statement in the Introduction to make this point much clearer.

Reviewer Point 1.4 *The proposed model has been validated on a subset of six channels only. It*

is not clear how these six channels have been selected from the 320 generated from the model and how they were matched with the positions on the muscle of the experimentally recorded EMG channels.

REPLY:
Thank you for highlighting the lack of a detailed description of the channel selection. We chose only six channels as we wanted to replicate the experimental settings and compare the synthetic data to the experimental recordings. In the experiments, the six bipolar electrodes were placed on the six target forearm muscles following the SENIAM guidelines [2], [3]. We simulated a total of 320 channels that are mounted uniformly around the forearm. Six channels were selected for each subject by matching the coordinates between the experimental electrodes and the simulated electrodes. We also checked the amplitudes of the selected channels during specific movements. For example, the signals collected by channels placed on the flexors should have a higher amplitude during wrist flexion compared to the amplitude during wrist extension. We have now added the details to the Results at lines 416-423.

Reviewer Point 1.5 *How were the experimental electrodes positioned on the muscles? Previous studies have shown that small misalignments between the position of the electrodes and the location of the individual muscles may create large errors in the performance of the regression.*

REPLY:
This is a good spot, thank you. Six EMG sensors (Delsys Trigno Wireless, Delsys Inc.) were placed on the six forearm muscles according to SENIAM guidelines [2]. The six muscles include flexor carpi radialis (FCR), flexor digitorum superficialis (FDS), flexor carpi ulnaris (FCU), extensor carpi ulnaris (ECU), extensor digitorum (ED), and extensor carpi radialis longus (ECRL). We chose a proper positioning of each EMG sensor by physically palpating the muscle during sustained isometric contraction and visually confirming the EMG signal. We have added these details to the Results at lines 397-406.

We agree with the reviewer that misalignments between the electrodes and muscles happen and will affect the performance of joint angle regression accuracy. Crosstalk due to the volume conduction makes the regression more challenging. This is why simple regression methods, for example linear regression and support vector regression, generally perform worse than more

complex data-driven or model-based methods [3]. This explains why there are variances in the regression performance across trials even for one subject, which we discussed in the Discussion in the revised manuscript. This also relates to the general reasoning behind building such forward models. We hope that when subject-specific anatomies are included, the proposed method can be used to simulate crosstalk and the distribution of electrical potentials during arbitrary movements. This will be useful to guide the electrode configuration and explain experimental results.

Reviewer Point 1.6 *The proposed model should be validated using a high-density dataset in combination with motor unit decomposition to compare the simulated variation in MUAP shapes with the ones estimated experimentally.*

REPLY:
We thank the reviewer for this insightful comment. Validation by comparing the decomposed MUAPs from high-density EMG and the synthetic MUAPs during dynamic movements is important but tricky at this stage. The challenges come from both predicting MUAPs from real data and generating synthetic MUAPs that exactly match the real MUAPs.

Current decomposition algorithms have only been validated for isometric contractions while several studies attempt to predict the changing motor unit filters during dynamic muscle contractions [4], [5]. MUAPs during dynamic movements could potentially be estimated by concurrently recording the intramuscular and surface high-density EMG signals with the assistance of ultrasound probes to place and record the intramuscular electrodes. The primary issue with this method is the consistent detection of the same motor unit across angles. In addition, it is not possible at this time to measure experimentally the bulk of the physiological parameters that characterise the MUAP generation. These two challenges make it difficult to compare the simulated variations in MUAP with the experimental ones. We hope the promise offered by the proposed model could stimulate the studies in these two directions. We have added this limitation and future work at the end of Discussion in the revised manuscript.

Reviewer Point 1.7 *Figure 8 reported the comparison between the regressions performed with the simulated and experimental signals. However, the comparison should be performed using multiple repetitions and subjects.*

REPLY:

We have now added the analyses and discussion of the regression performance over multiple simulation runs and six subjects to the Results. Detailed responses can be found in the reply to Point 1.1.

Reviewer Point 1.8 *Joint angle signals have very slow frequency components; therefore, the correlation coefficients with such slow signals may have a quite large lower bound. For these reasons, the authors should shuffle their dataset to estimate this lower bound and demonstrate that their results are significantly higher than that confidence level.*

REPLY:

Thank you for this insightful comment. The joint angles collected during the experiments changed slowly indeed, with the maximum frequency below 0.5 Hz. We agree with the reviewer that a lower bound should be estimated as a baseline that can be compared with the current reports. We estimated the lower bound in the following way. The ground truth joint angles from the testing set were shuffled and compared with the regressor's predictions. An extreme case is that if the joint angles are constant, shuffling the labels of joint angles will not affect the prediction accuracy. The random shuffling was repeated ten times for each trial.

The PCC values between the predicted joint angles and the shuffled experimental joint angles have a large variance and contain both positive and negative values, resulting in an averaged PCC around zero (-0.14 ± 3.68). We used the Student's t-test to compare the means between the two groups. The PCC between the predictions and the ground truth joint angles is significantly higher than the PCC between the prediction and the randomly shuffled angles ($p < 0.005$). We have added the comparison and the analyses to the Results at lines 444-453.

Reviewer 2

This paper proposes to create synthetic EMG that is compatible with muscle mechanics and motor unit function, taking into account the volume conduction of activation patterns through tissue on its way to the electrodes. As such, it is a very good first attempt towards generation of EMG in simulation, which has not yet been validated against a ground truth.

Major Points

Reviewer Point 2.1 *Figure 1: Based on Figure 1, the authors synthesize the EMG signals for the mentioned signals using the outputs from the simulated motion in OpenSim.*

- *Have the authors examined the difference between the EMG signals recorded experimentally in the real world and the synthesized signals? If yes, how different are these signals? What are the metrics to study these differences?*
- *What is the neural drive? Line 289-311 code block 2*
- *What are the "other parameters".*

REPLY:

We thank the reviewer for the insightful comments.

- Currently, it is difficult to examine the difference between the experimentally recorded EMG signals and the synthetic signals. The primary reason is that it is not possible at this time to measure experimentally most of the physiological parameters that contribute to EMG generation. Therefore, the synthetic EMG data cannot match the experimental settings exactly. In our work, we simulated data for six subjects during wrist/MCP flexion/extension movements. Even the error accumulated from the three modules, e.g., the forearm anatomy of each subject deviates from the anatomy used to train BioMime and the neural inputs to the motor unit pools come from the normalised experimental data, we still see high agreement between the synthetic data and the real data in some trials' simulations. We evaluated the similarity between the synthetic data and the real data by calculating the Pearson correlation coefficient (PCC) between the Root Mean Square (RMS) of these signals. The results showed that the PCC varies across channels. For channels that collect signals from extensor digitorum (ED) and extensor carpi radialis longus (ECRL), which are empirically easier to find by palpation, the PCC is above 0.70 for wrist regression and above 0.90 for MCP regression. For the slender muscles that are difficult to separate from others, the PCC is

lower, e.g., 0.40 for palmaris longus (PL) muscle in wrist angle regression. We have added the analyses to the Results at lines 548-554.

- Neural drive is the same as the neural input to the motor unit pool that determines the active status and the firing pattern of the motor unit. We apologise the expression is ambiguous here. We have unified the expressions to "neural input" in the revised manuscript. In the second code block, the variable 'ext' is a ramping neural input that increases from 0.0 to 1.0.
- The other parameters include the current source propagation velocity and the motor unit depth that can be estimated from muscle fibre length under constant muscle volume assumptions. We have added this detail to the caption of Figure 1 in the revised manuscript.

Reviewer Point 2.2 *Muscle models: The muscle models used by OpenSim and the Fuglevand model, Leaky Integrate and Fire (LIF) have multiple known limitations. For example, a recent review by Herzog emphasizes those for the Hill-Type model. While the authors are providing a computational, modular tool that can be repurposed, it is critical that the authors mention this as a strong limitation of their results.*

Reviewer Point 2.4 *More importantly, there is a critical assumption that is problematic: Muscles are considered de-afferented and only responsive to the neural drive (muscle activation). Such proprioceptive, spinal and propriospinal circuits greatly affect the activity of the motor units. This needs to be mentioned and highlighted as a critical limitation.*

Reviewer Point 2.5 *Similarly, it is important to highlight the critical limitation of how to handle passive lengthening and shortening in real limbs when the source neural drive to a muscle is not known.*

Reviewer Point 2.6 *The points made in 1—5 above are critical to the proposed use as "Test-bed for Cause-Effect Studies and Data Augmentation for Regression Analysis". This needs to be mentioned and the utility for this purpose toned down at this point.*

Reviewer Point 2.11 *Line 98: Assuming tendons to be rigid at constant slack length in musculoskeletal models can lead to several errors, including inaccurate force transmission, misrepresentation of muscle length changes, incorrect muscle activation patterns, inadequate representation of energy dynamics, and limited accuracy in predicting joint kinematics. It could be helpful to mention the errors introduced by this assumption.*

REPLY:

Thank you for the important comments. We gathered these five comments together as they all relate to the intrinsic limitations and assumptions taken in the various models integrated in NeuroMotion.

We agree with the reviewer that more details should be provided on the intrinsic limitations of the various models integrated in the NeuroMotion framework and that we should be more specific on how these limitations aggregate in the process of EMG generation using NeuroMotion. We have substantially revised the part of the Discussion that deals with limitations at lines 567-616. We have now discussed how the limitations intrinsic to the Hill-type muscle modelling and the Static Optimisation Tool in OpenSim affect the accuracy of muscle activation/neural inputs estimation in response to Points 2.2 and 2.4. We have added more details to the Section "Test-bed for Cause-Effect Studies and Data Augmentation for Regression Analysis" and have added the limitations that affect the results to the Discussion in response to Point 2.6. We have discussed the impact of assuming the rigid tendon and constant slack length of the musculoskeletal ARMs model on predicting muscle fibre lengths during movement in response to Point 2.11. In response to Point 2.5, NeuroMotion is developed for simulating surface EMG signals during voluntary movement, so it does not support simulations under passive movements. We have rephrased sentences in the Introduction to make this point much clearer.

Reviewer Point 2.3 *The system at this point is not compared to ground truth data, which is an important and critical limitation that needs to be addressed.*

REPLY:

We thank the reviewer for this important comment. As in the response to Point 1.6 from the first reviewer and the response to Point 2.1, it is currently challenging to compare the simulated data with the experimental data directly. The major challenge is that we cannot experimentally measure the factors that affect surface EMG generation. In this work, we compared the RMS features of the simulated EMG signals with the features of the experimental signals, even though there are mismatches between the forearm anatomy, neural inputs, and the MSK model. We surprisingly found that the RMS of the simulated signals for some subjects and trials aligned well with the experimental data. This is inspiring, as we would expect there will be a higher agreement when more factors are matched, e.g., by customising the BioMime model and the MSK model and by estimating the neural inputs to each muscle using invasive recordings. We have now added this

limitation to the end of the Discussion.

Reviewer Point 2.7 *Lines 155-157: As the text mentions, the toolbox incorporates two distinct neuron models (the classical Fuglevand's model and Leaky integrate and fire LIF). This applies to the Hill-type model as well.*

- *Conducting a comparative analysis of results obtained from each model module could offer valuable insights into their respective performance and applicability. For example, a simple Izhikevich neuron is a popular alternative to the LIF.*
- *Additionally, it would be beneficial to delve into more nuanced considerations regarding the practical utility of each model module, providing a comprehensive exploration of their specific use cases, advantages, and potential limitations.*
- *Such an examination would not only facilitate a deeper understanding of the toolbox's capabilities but also guide users in selecting the most suitable neuron model based on the specific requirements of their research or applications.*

REPLY:

We thank the reviewer for this very important comment. A comprehensive comparison among motor unit pool models is not the focus of this paper but will be investigated in the future. In this paper, we incorporate these two models into NeuroMotion and encourage users to choose their preferred one. For example, the user may prefer using the classical Fuglevand's model as it is more conceptually simple with fewer parameters and has been widely used in simulation works. The LIF model might be preferred in some cases, as it provides a more physiological way to predict the discharge activities based on the physiological dynamics of motoneuron membrane charging and discharging and allows finer control of motoneuron properties by providing more controllable parameters. The user could also add their self-developed models, for example the cited Izhikevich neuron model, into NeuroMotion.

Both Fuglevand's model and LIF model follow important physiological laws, e.g., the onion skin scheme and Henneman's size principle to estimate physiological properties and discharge activities of the motor units within each pool. The cohort of LIF models has been validated against experimental data by validating the predicted neural drive at the muscle scale and validating the individual MU discharges in a leave-one-out cross-validation procedure [6]. In contrast, the spiking activities output by Fuglevand's model were not directly validated in that paper [1]. We

have added these brief comparisons between the two models and basic suggestions on choosing a proper model into the Methods. We have also discussed the limitations of the two models in the new version of the manuscript.

Reviewer Point 2.8 *Line 147: The authors mention that the bulk of variations of the MUAPs are explained by the seven parameters; what is the basis for that? Is there a measurement or reasoning behind that?*

REPLY:
We chose the seven parameters by referring to the classical modelling references [7], [8], [9]. Physiological parameters that affect the MUAPs were first investigated by using analytical models, of which the parameters are easier to change compared with the numerical models. Table I in [7] summarised a list of parameters of the analytical models. Figure 1 in [7] displayed the parameters that shape one MUAP when straight fibres are distributed in parallel within an analytical volume conductor. The only parameter in Table I that affects MUAP waveforms and differs across motor units, muscles, and subjects but is not included in the seven parameters is the electrode-related parameter, which is automatically defined by the motor unit location and the electrode locations. We have added the references to the Methods.

Reviewer Point 2.9 *BIOMIME: Figure 2: The authors are using an AGNN in order to predict the MUAPs. The AGNN is conditioned to specific parameters.*

- *First, are the specified conditions the same as the seven supported parameters? If yes, do you train the pre-trained model using the specific conditions from the subject?*
- *In general, do you train the model at all? Or you just feed the samples and condition into the model for the inference mode?*
- *The figure is incomplete as it does not show how the signals of the discriminator are used.*

REPLY:

- The specified conditions are the same as the seven parameters. We trained BioMime on a dataset that was generated by an advanced numerical model [10]. The numerical model contains one forearm anatomy from a database but not from any of the six subjects in our case studies. We constructed the dataset by discretising the specified conditions and

traversing the conditions to generate MUAPs. Detailed description can be found in [11].

- BioMime is only used for inference in NeuroMotion. There is no need to retrain BioMime unless the user wants to customise their BioMime model. BioMime takes in the physiological parameter changes (muscle fibre length, current source conduction velocity, and motor unit depth) and outputs MUAPs during the dynamic movements.
- Figure 2 shows the training process of BioMime. During training, the generator and the discriminator are trained alternatively by competing with each other. The discriminator takes in a MUAP signal and a vector of the seven specified conditions and outputs whether the input MUAP is from real data and whether the input MUAP matches the conditions (True/False in Figure 2). Detailed algorithms and training pipeline can be found in [11]. BioMime can also be replaced by the other models that take in changing physiological parameters and output dynamic MUAPs.

Reviewer Point 2.10 *Lines 365-367: Authors are showing the similarity of the MUAPs in one muscle and the differences with the ones from other muscles. But this is not surprising, simply a quality control step, Figure 6. What is the point that is being made? To me, the important issue to discuss is the source of off-diagonal signals in the within-muscle comparison.*

REPLY:

We agree with the reviewer that the comparison in Figure 6 is a quality control step. Figure 6 shows the similarity and dissimilarity between MUAPs within and across muscles. We wanted to show two points here.

- MUAPs from different muscles have larger variances (nRMSE $>$ 0.02) than MUAPs from the same muscle. MUAPs from the two heads of the FCU muscle are more similar than MUAPs within FCU and a more distant muscle ECRB.
- MUAPs within the same muscle (off-diagonal signals) could have similar waveforms since multiple combinations of physiological parameters can contribute to similar MUAPs and different MUAPs may overlap in their territories due to the small muscle volume in the forearm.

Both of the two points are consistent with physiology. We have clarified these two points in the Results.

Minor Points

Reviewer Point 2.12 *Line 141: You mention that you are only using the generator (as it is in inference mode), however, the discriminator plays a pivotal role in assessing the realism of synthetic data compared to real counterparts. The discriminator's confidence scores, ROC and precision-recall curves, confusion matrix, and standard statistical metrics offer comprehensive insights into the model's ability to distinguish between real and synthetic data. Leveraging these evaluation methods enables a thorough understanding of the generator's performance, facilitating continuous monitoring and refinement to ensure the generation of high-quality synthetic data.*

REPLY:

We agree with the reviewer that the discriminator plays an important role in assessing the realism of the synthetic data and the performance of the generator. The optimal status is that when the GAN model converges, the discriminator cannot differentiate between real and fake ones, which means we get a perfect generator. This is achieved by training the generator and the discriminator alternatively. Details of the training pipeline, cost functions, and validations of BioMime can be found in [11]. We thank the reviewer for this useful suggestion.

Reviewer Point 2.13 *Line 149: Authors mention that the toolbox supports both methods, is there any comparison between the two of them?*

REPLY:

We have compared the difference between a MUAP generated by sampling from the prior distribution and a MUAP generated by morphing from an existing MUAP in BioMime work (not shown in the main context) [11]. When we plotted the MUAPs in the latent space using the first two components of t-SNE, the MUAPs generated by sampling or by morphing were all located within the original data's distribution unless the specified conditions deviated from the original data.

Users may want to use the "morphing" mode when they have some existing MUAPs and only want to change the specified conditions of them. In the examples of the case studies in this work, we first sampled the latent representations of each MUAP from the prior distribution and then input the fixed latent and the changing physiological parameters to the decoder to produce

the dynamic MUAPs. We have added these details to the Methods.

References

[1] A. J. Fuglevand, D. A. Winter, and A. E. Patla, "Models of recruitment and rate coding organization in motor-unit pools," *Journal of Neurophysiology*, vol. 70, no. 6, pp. 2470–2488, 1993.

[2] H. J. Hermens, B. Freriks, C. Disselhorst-Klug, and G. Rau, "Development of recommendations for sEMG sensors and sensor placement procedures," *Journal of Electromyography and Kinesiology*, vol. 10, no. 5, pp. 361–374, 2000.

[3] J. Zhao, Y. Yu, X. Sheng, and X. Zhu, "Consistent control information driven musculoskeletal model for multiday myoelectric control," *Journal of Neural Engineering*, 2023.

[4] V. Glaser and A. Holobar, "Motor unit identification from high-density surface electromyograms in repeated dynamic muscle contractions," *IEEE Transactions on Neural Systems and Rehabilitation Engineering*, vol. 27, no. 1, pp. 66–75, 2018.

[5] M. Kramberger and A. Holobar, "On the prediction of motor unit filter changes in blind source separation of high-density surface electromyograms during dynamic muscle contractions," *IEEE Access*, vol. 9, pp. 103533–103540, 2021.

[6] A. H. Caillet, A. T. Phillips, L. Modenese, and D. Farina, "NeuroMechanics: Electrophysiological and computational methods to accurately estimate the neural drive to muscles in humans in vivo," *Journal of Electromyography and Kinesiology*, p. 102873, 2024.

[7] R. Merletti, L. L. Conte, E. Avignone, and P. Guglielminotti, "Modeling of surface myoelectric signals. I. Model implementation," *IEEE Transactions on Biomedical Engineering*, vol. 46, no. 7, pp. 810–820, 1999.

[8] D. Farina and A. Rainoldi, "Compensation of the effect of sub-cutaneous tissue layers on surface EMG: a simulation study," *Medical Engineering & Physics*, vol. 21, no. 6-7, pp. 487–497, 1999.

[9] D. Farina, L. Mesin, S. Martina, and R. Merletti, "A surface EMG generation model with multilayer cylindrical description of the volume conductor," *IEEE Transactions on Biomedical Engineering*, vol. 51, no. 3, pp. 415–426, 2004.

[10] K. Maksymenko, A. K. Clarke, I. Mendez Guerra, S. Deslauriers-Gauthier, and D. Farina, "A myoelectric digital twin for fast and realistic modelling in deep learning," *Nature Communications*, vol. 14, no. 1, p. 1600, 2023.

[11] S. Ma, A. K. Clarke, K. Maksymenko, S. Deslauriers-Gauthier, X. Sheng, X. Zhu, and D. Farina, "Conditional generative models for simulation of EMG during naturalistic movements," 2024.
