# Supplementary material for: NeuroMotion: Open-source platform with neuromechanical and deep network modules to generate surface EMG signals during voluntary movement
Source: PLoS Comput Biol. 2024 Jul 3;20(7):e1012257. doi: 10.1371/journal.pcbi.1012257 (PMC11251629; doi:10.1371/journal.pcbi.1012257)
Supplement: Attachment — Submitted filename: NeuroMotion_response_letter.pdf [file pcbi.1012257.s002.pdf]

Response to Reviewers' Comments

Dear Editors of PLOS Computational Biology,

We are very grateful for the suggestions and comments from the editors and reviewers concerning our manuscript entitled "NeuroMotion: Open-Source Simulator to Generate High-Density EMG Signals during Voluntary Movement" (PCOMPBIOL-D-23-01615). We thank you most gratefully for the option to revise this paper. We also thank the reviewers for the time they have taken to produce the critique. We have considered all comments very carefully and made modifications, which we hope will address the concerns raised by the reviewers.

In the following, the comments from the reviewers are itemised for clarity and are addressed in order. The replies include references to the lines where the revisions to the text can be found. In the attached revised manuscript, all additions have been highlighted in yellow.

Reviewer 1

The authors have done an excellent work in revising the manuscript. All my previous comments have been addressed.
REPLY:
Thank you for your kind recommendation.

Reviewer 2

I thank the authors for their detailed responses to the reviewers. I will leave it to R1 to assess the completeness of the revised version their comments. Considering those comments and mine (R2), I have some additional clarifications, and in the interest of time, I will only mention major ones.
Limitations of this study: Many of the concerns that were expressed were addressed in the methods and discussion. In addition, the revised MS now has a more comprehensive expanded limitations section that includes many of those issues. However, it is worth noting that the limitations are multiple and important.
Reviewer Point 2.1 *So, at the end of the day, does the evidence support the claims made in the title and abstract, which were not revised? After the great effort made by the reviewers and*

authors to create the revised version, my perspective is that, unfortunately, the evidence does not entirely support the claims made in the original manuscript. This is amply elaborated in the reviewer's comments and revised Limitations.

REPLY:

We agree with the reviewer that further revisions to the title and abstract are required. Accordingly, we have revised the manuscript following the reviewer's comments 2.3 and 2.4.

Reviewer Point 2.2 *The value of this work is the platform. The validity of the muscle physiology and EMG generation that are used in this first use-case are, as per the limitations, debatable and simply one of the options the field currently has. The authors need not limit the value of their work to the initial generative EMG work presented (which has good points but also many limitations). There is no need for the value of the work to be dependent on the details of this first use-case.*

REPLY:

Thank you for the insightful comment and suggestion. It is true that the proposed platform is the major contribution of this work, and each of the three sub-modules can be updated with the advancement in each field. As suggested by the reviewer in points 2.3 and 2.4, we have revised the title and abstract accordingly.

Reviewer Point 2.3 *I have much respect for the authors so, it is with humility and solely in the interest of time, that I point out what I believe the evidence supports (tracked changes as CAPS).*

- *Original Title (not edited in the revised version): "NeuroMotion: Open-source Simulator with Neuromechanical and Deep Network Models to Generate Surface EMG signals during Voluntary Movement".*
- *My commented one "NeuroMotion: Open-source PLATFORM with Neuromechanical and Deep Network MODULES to Generate Surface EMG signals during Voluntary Movement* This is important because the main and well-justified contribution is the modular computational platform. As to the results and validity of the specific initial EMG predictions, the Limitations listed clearly show this is simply a first use-case.

REPLY:

We have now changed the title to "NeuroMotion: Open-Source Platform with Neuromechanical and Deep Network Modules to Generate Surface EMG signals during Voluntary Movement", as suggested.

Abstract (not edited in the revised version)

Reviewer Point 2.4

- *Original text "...However, current simulation models of electromyography (EMG), a core physiological signal in neuromechanical analyses, are either limited in accuracy and conditions or are computationally heavy to apply. Here, we overcome these limitations by presenting NeuroMotion, an open-source simulator that allows a full-spectrum synthesis of EMG signals during voluntary movements...."*
- *My suggested text: "...However, current simulation models of electromyography (EMG), a core physiological signal in neuromechanical analyses, REMAIN either limited in accuracy and conditions or are computationally heavy to apply. Here, we PROVIDE A COMPUTATIONAL PLATFORM TO ENABLE FUTURE WORK TO overcome these limitations by presenting NeuroMotion, an open-source simulator that CAN MODULARLY TEST A VARIETY OF APPROACHES TO THE full-spectrum synthesis of EMG signals during voluntary movements...."*
- *Original text: "... NeuroMotion is comprised of three modules."*
- *My suggested text: "WE DEMONSTRATE NeuroMotion USING three SAMPLE modules."*
- *Original text: "...NeuroMotion is the first full-spectrum EMG generative model to simulate human forearm electrophysiology during voluntary hand, wrist, and forearm movements. ...."*
- *My suggested text: "...IN THIS WAY, NeuroMotion WAS ABLE TO GENERATE full-spectrum EMG FOR A FIRST USE-CASE of human forearm electrophysiology during voluntary hand, wrist, and forearm movements. ...."*
- *Original text: "...We expect this full-spectrum model will complement experimental approaches and facilitate neuromechanical research."*
- *My suggested text: "...We expect this MODULAR PLATFORM will ENABLE VALIDATION OF GENERATIVE EMG MODELS, complement experimental approaches and EMPOWER neuromechanical research."*

*These edits to the Abstract are super critical as the abstract needs to reflect the main defensible (and valuable) computational contributions, and (implicitly) acknowledge the many limitations at the physiological levels. Making these changes—**and toning down the claims in the Discussion to align with this**—allows for a very valuable MS without the need to do many, if any, revisions to the Methods or Results.*

REPLY:

We thank the reviewer for the very detailed suggestions to the abstract. We fully agree on all revisions and we thank for providing us with such a clear suggestion. We have implemented all revisions as suggested by the reviewer.

Reviewer Point 2.5 *In the modeling community, many are now moving away from OPENSIMM un favor of using MuJoCo as the physics and graphics engine. It is a more computationally efficient platform that reads SIMM-format XML files. MuJoCo may, incidentally, be a better platform for the computationally demanding approach the authors use. Readers should be made aware of this movement in the field. For example, MyoSim.*

Wang, H., Caggiano, V., Durandau, G., Sartori, M. and Kumar, V., 2022, May. MyoSim: Fast and physiologically realistic MuJoCo models for musculoskeletal and exoskeletal studies. In 2022 International Conference on Robotics and Automation (ICRA) (pp. 8104-8111). IEEE.

Please add other citations as well.

REPLY:

We have added MuJuCo as one of the widely used simulation platforms in the introduction and have added the references as well.

Reviewer Point 2.6 *One of my original comments was the importance of including subcortical circuits. This was just mentioned in the Limitations but with no references.*

- *I suggest this change of wording that add reflexes explicitly ("The Static Optimisation method also does not consider the concurrent dynamics of the proprioceptive, spinal and propriospinal circuits AND REFLEXES during voluntary movement in the computation of muscle activations, and hence, of neural inputs.")*
- *References should be included. A good reference is below, but please add others.*

Song, S., Kidziński, Ł., Peng, X.B. et al. Deep reinforcement learning for modeling human locomotion control in neuromechanical simulation. J NeuroEngineering Rehabil 18, 126 (2021). https://doi.org/10.1186/s12984-021-00919-y

REPLY:

We have revised the sentence and added six references, including the reference provided, over the 1995-2021 time period, to give examples of how subcortical circuits can be considered and modelled for the computation of the neural input in neuromuscular and musculoskeletal simulations.

Reviewer Point 2.7 *It remains hugely problematic to use muscle fiber length (which can be passive) as a means to estimate EMG—especially when the model of the muscle is deafferented, as per my prior review. This should be mentioned in the limitations with references.*

REPLY:

We have revised the manuscript, in the same paragraph as for Reviewer Point 2.6, to comment on the limitation of using muscle fibre length to estimate EMG. We would like to raise that EMG is not estimated only from muscle fibre length in NeuroMotion, although we acknowledge that muscle fibre length and activation are somewhat coupled when obtained from static optimisation. Notably, a passive muscle that passively changes muscle fibre length would have different MUAP shapes but no spike train activity, and therefore no predicted EMG activity.

Reviewer Point 2.8 *So sorry, but in response to one of my comments, the justification for ignoring tendon length and elasticity as "theoretically acceptable" is much too facile. Zajac himself used forearm muscles as an example of where the properties of these long tendons are important. It is OK to make assumptions, and also OK to say they are done in the interest of computational convenience—but there is no need to say it is physiologically correct.*

Zajac, Felix E. "How musculotendon architecture and joint geometry affect the capacity of muscles to move and exert force on objects: a review with application to arm and forearm tendon transfer design." The Journal of hand surgery 17.5 (1992): 799-804.

REPLY:

We thank the reviewer for this comment. We removed the sentence that suggested physiological

accuracy, acknowledged the simplification for computational convenience, and added the suggested reference to complete our discussion.
